# Near-infrared light-heatable platinum nanozyme for synergistic bacterial inhibition

**DOI:** 10.3389/fbioe.2024.1355004

**Published:** 2024-01-16

**Authors:** Xue Li, Weisheng Zhu, Yuan Zhou, Nan Wang, Xiangfan Gao, Suling Sun, Mengting Cao, Zhijun Zhang, Guixian Hu

**Affiliations:** ^1^ Institute of Agro-product Safety and Nutrition, Zhejiang Academy of Agricultural Sciences, Hangzhou, China; ^2^ Key Laboratory of Information Traceability for Agricultural Products, Ministry of Agriculture and Rural Affairs of China, Hangzhou, Zhejiang, China; ^3^ Key Laboratory of Surface & Interface Science of Polymer Materials of Zhejiang Province, School of Chemistry and Chemical Engineering, Zhejiang Sci-Tech University, Hangzhou, China; ^4^ Department of Pharmacy, Taihe Hospital, Hubei University of Medicine, Shiyan, China; ^5^ College of Pharmacy, Hubei University of Traditional Chinese Medicine, Wuhan, China; ^6^ Shengzhou Innovation Research Institute of Zhejiang Sci-Tech University, Shengzhou, China

**Keywords:** nanozyme, metal–organic framework, photothermal effect, reactive oxygen species, bacterial inhibition

## Abstract

The development of non-antibiotic strategies for bacterial disinfection is of great clinical importance. Among recently developed different antimicrobial strategies, nanomaterial-mediated approaches, especially the photothermal way and reactive oxygen species (ROS)-generating method, show many significant advantages. Although promising, the clinical application of nanomaterials is still limited, owing to the potential biosafety issues. Further improvement of the antimicrobial activity to reduce the usage, and thus reduce the potential risk, is an important way to increase the clinical applicability of antibacterial nanomaterials. In this paper, an antimicrobial nanostructure with both an excellent photothermal effect and peroxidase-like activity was constructed to achieve efficient synergistic antimicrobial activity. The obtained nano-antimicrobial agent (ZIF-8@PDA@Pt) can not only efficiently catalyze the production of ROS from H_2_O_2_ to cause damage to bacteria but also convert the photon energy of near-infrared light into thermal energy to kill bacteria, and the two synergistic effects induced in a highly efficient antimicrobial activity. This study not only offers a new nanomaterial with efficient antibacterial activity but also proposes a new idea for constructing synergistic antibacterial properties.

## 1 Introduction

Bacterial infection is a serious threat to global health, which is estimated to be the second leading cause of death worldwide, accounting for one in eight of all deaths in 2019 (Ikuta et al., 2022). Antibiotics, also known as antimicrobials, are the most commonly used treatment for bacterial infections. Antibiotics are a class of secondary metabolites with antipathogenic or other activities produced by microorganisms or higher plants and animals, which have shown high activity against bacterial infections. However, bacteria develop resistance to antibiotics over time, and the problem of bacterial resistance is growing due to the misuse of antibiotics. It is estimated that more than 1.2 million people—and potentially millions more—died globally a year as a direct result of antibiotic-resistant bacterial infections, according to the most comprehensive estimate, to date, of the AMR) (Murray et al., 2022). Unfortunately, the rate of development of new antibiotics is much slower than the rate of emergence of bacterial resistance. In addition, the use of antibiotics for anti-infective therapy is prone to side effects such as liver damage and kidney injury. Thus, the development of novel antimicrobial strategies to reduce the use of antibiotics is of great clinical importance.

Many different new antimicrobial strategies have been reported in recent years (Sun et al., 2020; Niu et al., 2021a; Niu et al., 2021b; Du et al., 2021; Zhang et al., 2021; Li et al., 2022a; Li et al., 2022b; Fu et al., 2022; Guo et al., 2022; Wang et al., 2023; Wu et al., 2023). Among them, nanomaterial-mediated antimicrobial strategies show more significant advantages, including easy material synthesis, being less susceptible to bacterial resistance, and a high bacterial killing efficiency. In these strategies, nanomaterials usually play the role of mediators of energy or material conversion. Typically, nanomaterials can convert the energy of external physical stimuli into thermal energy that can directly damage the bacterial structure (Liu et al., 2022a; Hu et al., 2022). The photothermal antimicrobial strategy is such an energy conversion-based bacterial disinfection method, which is favored for its high killing efficiency and spatial and temporal controllability (Han et al., 2020). The photothermal effect is a property of materials that can harvest and transfer the photon energy from irradiated light to heat energy, which is promising for wide applications (Wang et al., 2019; Wu and Yeow, 2022; Zeng et al., 2022; Fan et al., 2023; Huang et al., 2023; Jiao et al., 2023; Yue et al., 2023; Zhao et al., 2023; Zhu et al., 2023). In the photothermal antimicrobial strategy, nanomaterials with photothermal conversion efficiency, such as graphene, Fe_3_O_4_, polydopamine (PDA), and gold nanostructures, can efficiently convert the photon energy of near-infrared light into thermal energy to achieve efficient killing of different types of bacteria (Niu et al., 2018; Wang et al., 2022). In addition, nanomaterials with enzyme-like activity can convert endogenous or exogenous chemicals into high-energy reactive oxygen species (ROS) to cause bacterial casualties (Chen et al., 2018; Liu et al., 2022b; Dorma Momo et al., 2022). For example, nanoenzymes such as graphene oxide and carbon dots can catalyze the production of ROS from highly expressed or exogenous H_2_O_2_ at the site of bacterial infection to cause the destruction of bacterial nucleic acids and proteins and thus lead to bacterial death. Nanomaterial-mediated antimicrobial strategies undoubtedly offer unlimited possibilities for replacing the use of antibiotics (Makabenta et al., 2021; Xie et al., 2023). Nevertheless, the clinical application of nanomaterials is still very limited at present as the biosafety issues of their clinical application are yet to be fully evaluated. Further improvement of antimicrobial activity to reduce the usage, and thus reduce the potential risk, is an important way to increase the clinical applicability of antibacterial nanomaterials.

**SCHEME 1:**
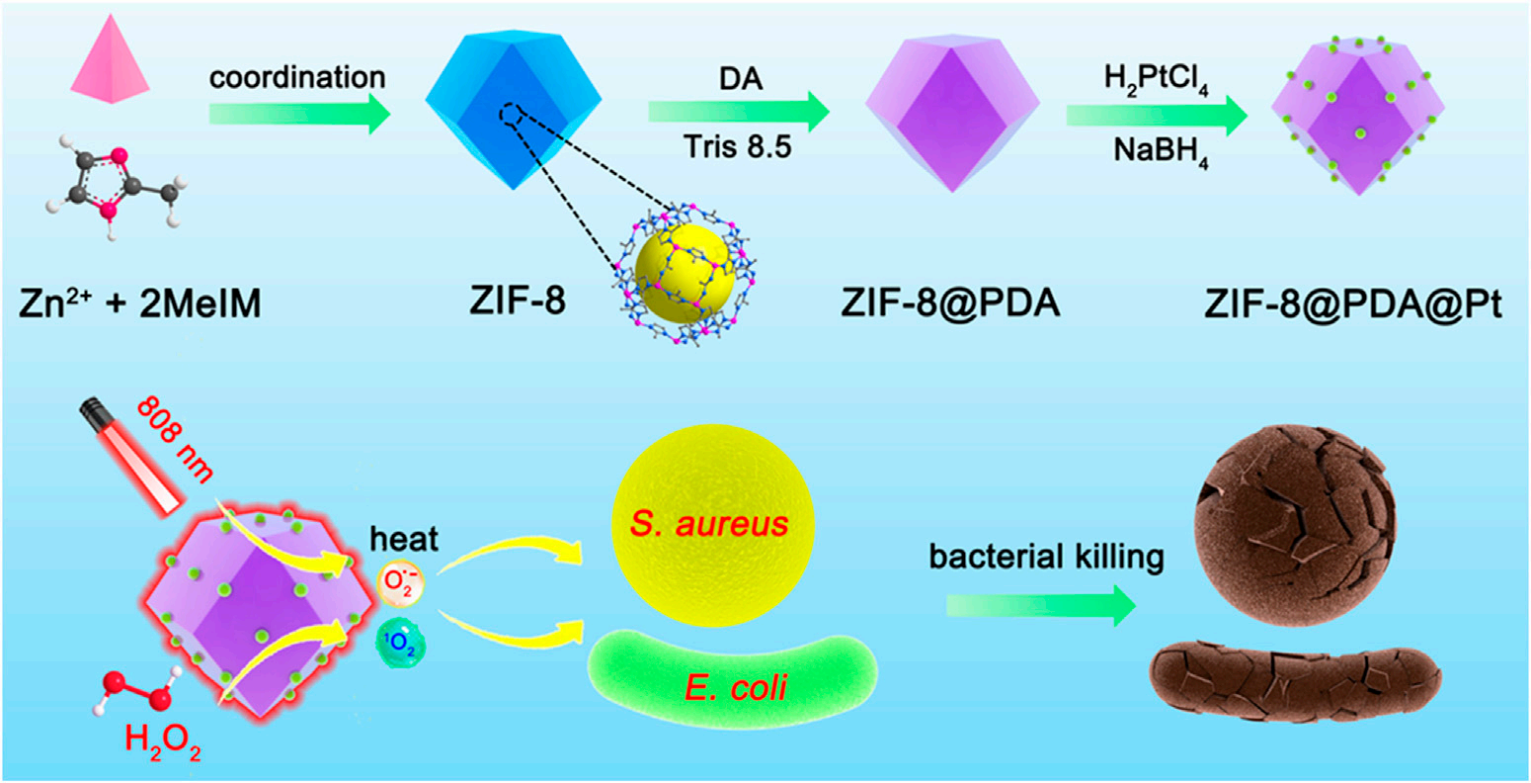
Schematic illustration of the synthesis of ZIF-8@PDA@Pt and the principle of bacterial killing.

In this paper, an antimicrobial nanostructure with both the photothermal effect and peroxidase-like activity was constructed to combine the photothermal antimicrobial and free radical antimicrobial strategies to achieve efficient synergistic antimicrobial activity (Scheme 1). ZIF-8, a frequently used metal–organic framework (MOF) in biomedical research, was chosen as the nanocarrier. A PDA layer with an excellent photothermal effect was encapsulated on the surface of ZIF-8 by *in situ* polymerization, and then, platinum nanoparticles (PtNPs) with peroxidase-like activity were allowed to grow on the PDA layer. The obtained nano-antimicrobial agent (ZIF-8@PDA@Pt) can not only efficiently catalyze the production of ROS from H_2_O_2_ to cause damage to bacteria but also convert photon energy of the near-infrared light into thermal energy to kill bacteria, and the two synergistic effects induced in a highly efficient antimicrobial activity.

## 2 Materials and methods

### 2.1 Synthesis of ZIF-8@PDA@Pt

ZIF-8 was synthesized through a specific coordination reaction between zinc ions and 2-methylimidazole. In a typical experiment, 1.069 g Zn(NO_3_)_2_·6H_2_O was dissolved in 15 mL of DMF-MeOH mixture (v/v = 4:1) as solution **1**, 1.161 g of 2-methylimidazole was dissolved in 10 mL of DMF-MeOH (v/v = 4:1) as solution **2**; then, solution **1** was added to solution **2** and kept stirring vigorously overnight. Subsequently, the milky product was collected by centrifugation at 14,000 rpm for 10 min, rinsed with methanol and ultrapure water two times, and then dried at 80°C overnight to obtain ZIF-8 for subsequent experiments.

ZIF-8@PDA was attained by a self-polymerization reaction of dopamine. A measure of 20 mg ZIF-8 we prepared was dissolved in the Tris-HCl buffer solution (pH 8.5, 50 mM) with the assistance of sonicating for 2 min; then, 10 mg dopamine was added to the above solution, and the mixture was violently stirred for 90 min. Thereafter, the obtained black mixture was collected by centrifugation at 14,000 rpm for 10 min, washed with water three times, and eventually concentrated to 2 mL for later use.

Subsequently, ZIF-8@PDA@Pt was gained by an *in situ* reduction method using ZIF-8@PDA as a precursor. A measure of 2 mL of ZIF-8@PDA we prepared previously was added to 7,740 μL ultrapure water and sonicated for 2 min; then, 260 μL of H_2_PtCl_4_ (19.2 mM) was added and sonicated for 5 min; in a rapid subsequence, 500 μL of the refresh NaBH_4_ solution (1 M) was added to the above solution under drastic magnetic stirring for 1 h. Finally, the mixture was collected by centrifugation at 14,000 rpm for 10 min and ultimately washed with ultrapure water three times to obtain ZIF-8@PDA@Pt.

### 2.2 Measurement of peroxidase-mimic activity

The peroxidase-mimic activity of ZIF-8@PDA@Pt was measured by a classical chromogenic reaction using TMB as the substrate. In brief, 5 μL TMB (80 mM), 50 mL H_2_O_2_ (10 mM), and 50 μL ZIF-8@PDA@Pt (0.2 mg mL^−1^) were added to the phosphate buffer solution (pH 5.0, 20 mM) with a total volume of 0.5 mL, respectively. Subsequently, the mixture was incubated for 5 min, and the absorbance at 652 nm was recorded simultaneously by an ultraviolet-visible spectrum. o-Phenylenediamine (OPD) was employed as a chromogenic substrate to further evaluate the peroxidase-mimic activity of ZIF-8@PDA@Pt. Typically, 5 μL OPD (50 mM), 50 mL H_2_O_2_ (10 mM), and 50 μL ZIF-8@PDA@Pt (0.2 mg mL^−1^) were added to the phosphate buffer solution (pH 5.0, 20 mM) with a total volume of 0.5 mL, respectively. Subsequently, the mixture was incubated for 5 min, and the absorbance at 425 nm was recorded simultaneously by an ultraviolet-visible spectrum.

### 2.3 Catalytic kinetic assay

The kinetic assay of the peroxidase-mimic activity of ZIF-8@PDA@Pt was conducted by changing the concentration of H_2_O_2_ or TMB. The reaction was performed under an optimized condition (pH 5.0, 37°C). Typically, 2 μL TMB (80 mM), H_2_O_2_ with different concentrations, and 20 μL ZIF-8@PDA@Pt (0.2 mg mL^−1^) were added to a phosphate buffer solution (pH 5.0, 20 mM) with a total volume of 0.2 mL; then, the absorbance at 652 nm was recorded in time at a time scanning window. The real-time velocity of the reaction was converted by the Beer–Lambert Law, A = εbC, where b is the path length (1 cm) and *ε* is the molar absorption coefficient of oxidized TMB (39,000 M^−1^ cm^−1^). Corresponding parameters of kinetics were calculated according to the Michaelis–Menten equation and Lineweaver–Burk plot:

$$V_{0}=V_{\max}\frac{\left[S\right]}{\left[S\right]+{\;K}_{m}},$$

$$\frac{1}{V_{0}}=\frac{{\;K}_{m}}{V_{\max}}\;\frac{1}{\left[S\right]}+\frac{1}{V_{\max}},$$

where *V*_*0*_ is the initial reaction rate and [*S*] is the concentration of the substrate (H_2_O_2_ or TMB).

### 2.4 Detection of ROS generation

Terephthalic acid (TA) was used as a fluorescent probe to validate the generation of •OH. Typically, TA (0.5 mM), H_2_O_2_ (2 mM), and ZIF-8@PDA@Pt (0.1 mg mL^−1^) were first mixed into PBS (pH = 5.0, 20 mM) and incubated in dark for 2 h, and then were taken for fluorescent spectra characterization. In addition, 1,3-diphenylisobenzofuran (DPBF) and 9,10-anthracenediyl-bis(methylene)-dimalonic acid (ABDA) were used as probes to assay O_2_^•−^ and ^1^O_2_, respectively. Specifically, DPBF (1 mM), H_2_O_2_ (2 mM), and ZIF-8@PDA@Pt (0.1 mg mL^−1^) were incubated together in PBS (pH = 5.0, 20 mM) for 5 min; meanwhile, the absorbance at 421 nm was recorded every 30 s. ABDA (0.05 mM), H_2_O_2_ (2 mM), and ZIF-8@PDA@Pt (0.1 mg mL^−1^) were incubated together in PBS (pH = 5.0, 20 mM) for a while and then were taken to measure the change of absorbance using UV spectra.

### 2.5 Determination of the photothermal property

The photothermal property of ZIF-8@PDA@Pt was determined using an 808-nm laser, and the real-time temperature was observed with a thermal imaging camera. In brief, ZIF-8@PDA@Pt aqueous dispersion with different concentrations (0, 20, 40, 60, 80, 100, and 120 ug mL^−1^) was radiated under 808 nm (1.0 W cm^−1^) for 5 min. Concurrently, the temperature of the ZIF-8@PDA@Pt aqueous dispersion was recorded every 30 s. Thereafter, the heating–cooling curves were obtained to calculate the photothermal conversion efficiency in accordance with below equations:

$$\eta =\frac{hS\;\left(T_{\max}\;‐{\;T}_{\text{surr}}\right)\;‐{\;Q}_{0}}{I\;\left(1\;‐{\;10}^{‐A_{808}}\right)},$$ (1)

$$\tau_{s}=\frac{m_{d}C_{d}}{hS},$$ (2)

$$Q_{0}=hS\;\left(T_{\max,\text{water}}\;‐{\;T}_{\text{surr}}\right),$$ (3)

where *η* is the photothermal conversion efficiency. The value of *τ*_*s*_ was obtained by linearly fitting the plot of the cooling time t versus the term -Lnθ, *hS* was obtained from Eq. 2, *m*_*d*_ is the mass of the solution (0.2 g), *C*_*d*_ is the heat capacity of water (4.2 J g^−1^°C^−1^), Q_0_ was obtained from Eq. 3, *T*_max_ is the equilibrium temperature (61.5°C), *T*_max, water_ is the maximum temperature of water (29.1°C), and *T*_surr_ is the surrounding ambient temperature (28.3°C). *I* is the incident light power (0.8 W cm^−2^), and A_808_ is the absorbance of the ZIF-8@PDA@Pt aqueous dispersion (120 μg mL^−1^) at 808 nm (0.17).

### 2.6 Antibacterial activity test

*Staphylococcus aureus* and *Escherichia coli* were selected as Gram-positive and Gram-negative model strains to estimate the antibacterial activity of ZIF-8@PDA@Pt, respectively. Mono colonies of *S*. *aureus* and *E. coli* on a solid agar plate were first transferred to 2 mL of the lysogeny broth (LB) medium and shaken at 37°C for 12 h at 150 rpm. Four different groups were set in this typical procedure, containing I: Control, II: H_2_O_2_, III: ZIF-8@PDA@Pt + H_2_O_2_, and IV ZIF-8@PDA@Pt + H_2_O_2_ + NIR. In addition, 0.3 was selected as the initial optical density of bacteria at OD_600 nm_. In addition, the final concentration of H_2_O_2_ and ZIF-8@PDA@Pt in the incubation system was 2 mM and 100 μg mL^−1^, respectively. All groups were incubated at 37°C for 20 min. Thereafter, the bacterial suspensions were moved to the solid medium by the spread plate method and cultured at 37°C for another for 12 h. Finally, all experimental groups were taken for imaging and counting. Each assay was performed in triplicates.

### 2.7 Live/dead bacterial staining

Bacterial suspensions treated with the different experimental conditions were first centrifuged for 5 min (4,000 rpm), followed by washing two times using 0.85% NaCl solutions. Afterward, the above bacterial suspensions were stained with SYTO 9 and propidium iodide and then observed with a fluorescent microscope.

### 2.8 Cell viability assay

HSF cells were performed for the biocompatibility assay of ZIF-8@PDA@Pt. At first, the HSF cells were cultured in a 96-well plate (8,000 cells per well) at 37°C for 24 h; then, ZIF-8@PDA@Pt with different concentrations (0, 25, 50, 100, 150, and 200 μg mL^−1^) were added to the above medium and incubated for another 24 h at 37°C. Thereafter, 10 μL of the MTT solution was added to every well. After 4 h, the medium was extracted and 150 μL of DMSO was added to every well. The cell viability was assayed using a microplate reader.

## 3 Results and discussion

### 3.1 Synthesis and characterization of ZIF-8@PDA@Pt

**FIGURE 1:**
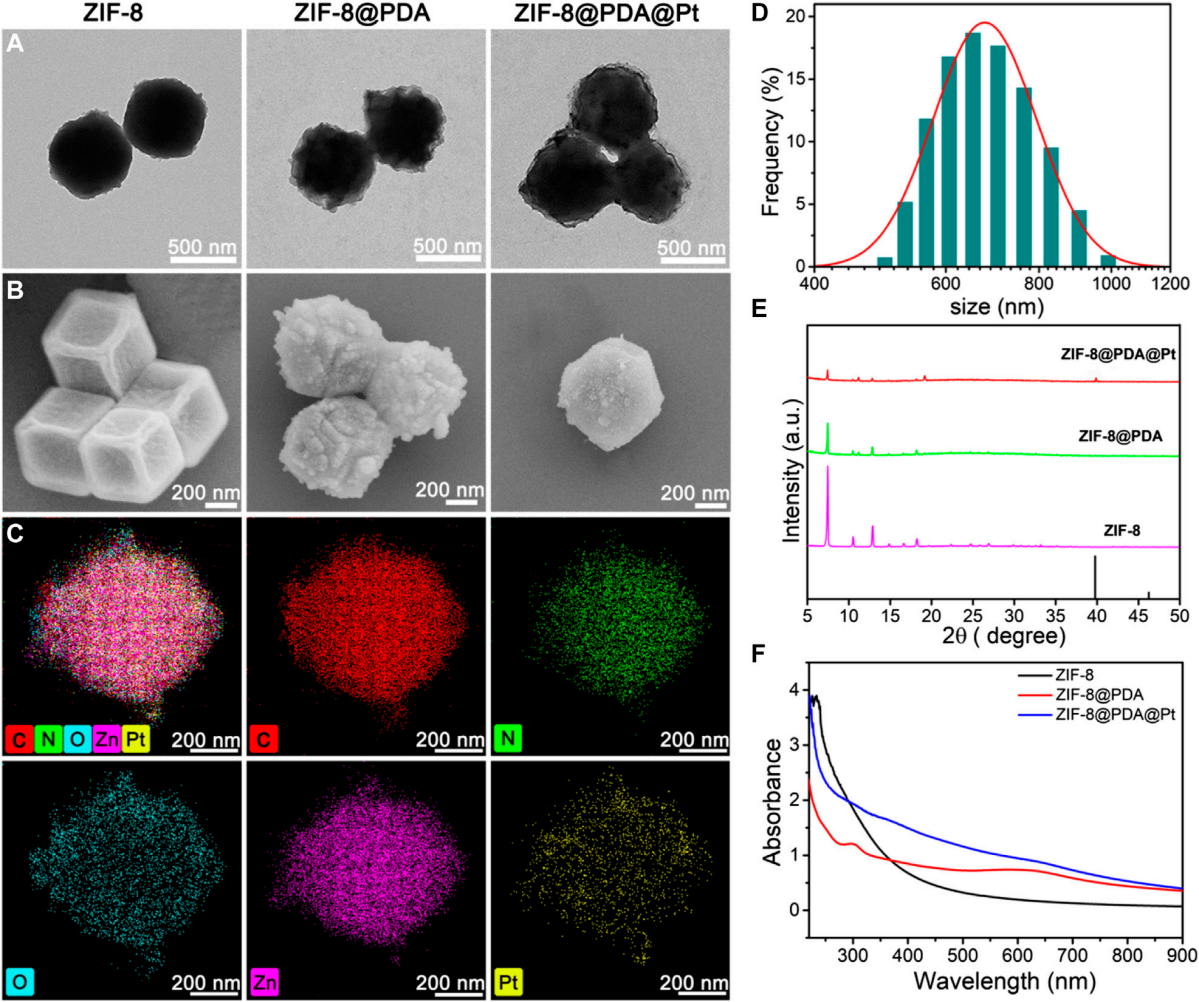
TEM **(A)** and SEM **(B)** images of the nanostructures, and the elemental mapping **(C)** and size distribution **(D)** of ZIF-8@PDA@Pt. XRD patterns **(E)** and UV-vis spectra **(F)** of these nanoparticles.

ZIF-8@PDA@Pt was prepared through *in situ* reduction of H_2_PtCl_4_ in the presence of a specific precursor obtained by modifying a layer of polydopamine on the surface of ZIF-8. To begin with, ZIF-8 was conducted by a specific coordination reaction between zinc ions and 2-methylimidazole. Afterward, a layer of PDA was modified on ZIF-8, relying on the self-polymerization reaction of dopamine under a weak alkaline environment to obtain ZIF@PDA. Eventually, Pt NPs were localized on ZIF@PDA by *in situ* reduction to obtain the nanozyme ZIF-8@PDA@Pt. TEM and SEM were primarily employed to characterize the morphology during the formation of ZIF-8@PDA@Pt (Figures 1A, B); it is clearly to be observed that ZIF-8 possesses the regular rhombic dodecahedral morphology while a thin layer at the edge of ZIF-8 was noticed after modifying polydopamine, verifying the formation of ZIF-8@PDA. In addition, it can be seen that ultra-small Pt NPs were distributed on ZIF-8@PDA. In addition, energy-dispersive X-ray spectrometry (EDX) mapping was recruited to characterize the elemental distribution of ZIF-8@PDA@Pt nanozyme (Figure 1C). The results presented the homogeneous distributions of elements C, N, O, Zn, and Pt in ZIF-8@PDA@Pt, demonstrating the successful loading of Pt NPs onto the ZIF-8@PDA platform. Meanwhile, ZIF-8@PDA@Pt exhibits a size of approximately 650 nm, according to the dynamic light scattering analysis (Figure 1D).

Additionally, in order to explore the chemical structure and composition, we executed X-ray diffraction (XRD), X-ray photoelectron spectroscopy (XPS), and UV-vis spectra. As illustrated in Figure 1E, the pattern of ZIF-8 exhibits several typical characteristic peaks at around 7.46°, 10.52°,12.87°, and 18.27°, ascribing to (001), (002), (112), and (222) crystal faces, respectively, which conforms its regular crystal structure. Of note, the main diffraction peaks of ZIF-8 remained after modifying PDA and loading Pt NPs. In particular, the XRD diffraction of ZIF-8@PDA@Pt presents an unmistakable peak at 39.5° in line with the (111) facet of the Pt crystal (JCPDS No. 04-0802), which further confirmed the successful preparation of ZIF-8@PDA@Pt. The UV-vis spectra were also measured and are presented in Figure 1F. It can be noted that two typical peaks at approximately 234 and 300 nm in ZIF-8 and ZIF-8@PDA assigned to the *π*-π* transition of the conjugated system from the imidazole structure and *n*-π* transition of polydopamine, respectively. Nonetheless, the peak at approximately 300 nm is not well-preserved in ZIF-8@PDA@Pt, which may be caused within the loading of Pt NPs. In addition, XPS was performed to characterize the element constitution of ZIF-8@PDA@Pt (Supplementary Figure S1). The element contents of C, N, O, Zn, and Pt are 53.08%, 9.55%, 25.03%, 10.72%, and 1.62%, respectively. More precisely, in the high-resolution spectrum of Pt 4f, the peaks located at 71.0, 72.2, 74.2, and 75.5 eV are ascribed to Pt^0^ 4f 7/2, Pt^4+^ 4f 7/2, Pt^0^ 4f 5/2, and Pt^4+^ 4f 5/2 binding energies, respectively, which are analogous to the majority of Pt NPs. Likewise, several typical peaks at approximately 284.5, 286.2, and 288.3 eV can be identified from the high-resolution C1s spectrum, which are assigned to C-C, C-N/C-O, and C=C groups, respectively. It can be observed that three peaks are located at 398.2, 399.3, and 400.7 eV from the N 1s spectrum, corresponding to pyridinic N, pyrrolic N, and graphitic N, respectively. Comparably, the curve-fitted O1s spectrum exhibits two peaks 531.3 and 533.1 eV, attributing to C-O and C-OH groups, respectively. In addition, the high-resolution Zn 2p spectra of ZIF-8@PDA@Pt present two peaks placed at 1022.3 and 1045.3 eV belonging to Zn 2p 1/2 and Zn 2p 2/3 of zinc oxide, respectively. In combination with the above results, it is believed that ZIF-8@PDA@Pt was successfully prepared.

### 3.2 Catalytic performance of ZIF-8@PDA@Pt

**FIGURE 2:**
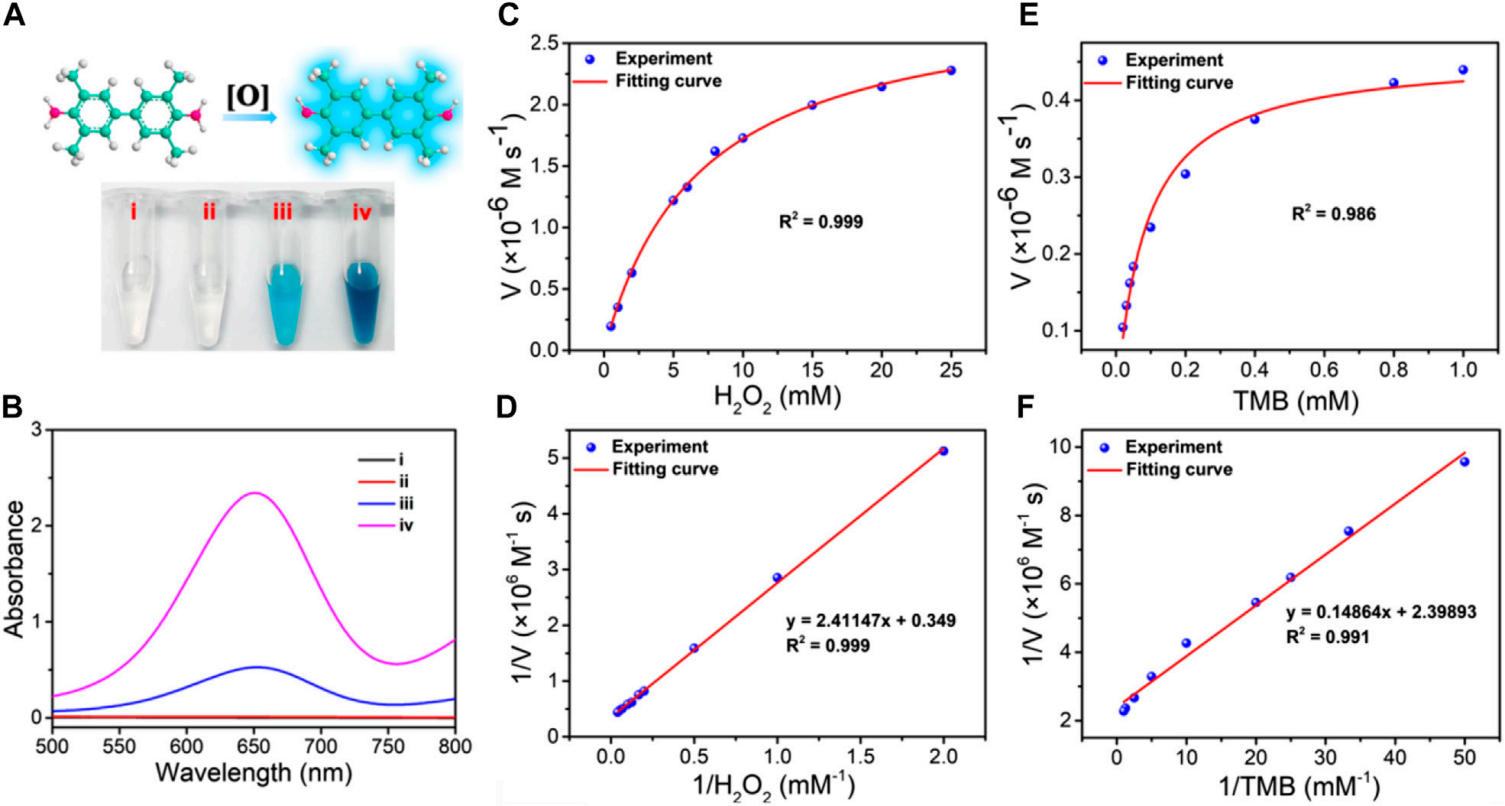
**(A)** Images and **(B)** UV-vis absorption spectra of the TMB solution under different conditions (i: TMB, ii: TMB + H_2_O_2_, iii: ZIF-8@PDA@Pt + TMB, and iv: ZIF-8@PDA@Pt + TMB + H_2_O_2_). **(C)** Michaelis–Menten curve under 0.8 mM TMB and various concentrations of H_2_O_2_. **(D)** Lineweaver–Burk plot for H_2_O_2_. **(E)** Michaelis–Menten curve under 1 mM H_2_O_2_ with various concentrations of TMB. **(F)** Lineweaver–Burk plot for TMB.

After successful synthesis of the ZIF-8@PDA@Pt nanoparticles, we then investigated its peroxidase (POD)-like activity using 3,3′,5,5′-tetramethylbenzidine (TMB) as the chromogenic substrate and H_2_O_2_ as the catalysis substrate. As shown in Figure 2A, without ZIF-8@PDA@Pt, H_2_O_2_ cannot cause any color change in the TMB solution. However, in the presence of ZIF-8@PDA@Pt and H_2_O_2_, the color of the TMB solution significantly changed to dark blue (Figures 2A–2iv), indicating the strong POD-like activity of ZIF-8@PDA@Pt. In this catalytic system, ZIF-8@PDA@Pt may catalyze H_2_O_2_ to produce ROS, which can oxidize TMB to generate blue color. Notably, without H_2_O_2_, the nanozyme ZIF-8@PDA@Pt can also cause an obvious change in the color of the TMB solution (Figures 2A–2iii), demonstrating that the nanozyme also possesses oxidase-like activity. The enzyme-like activity of ZIF-8@PDA@Pt was also demonstrated from the absorbance change of the TMB solution (Figure 2B). To further evaluate the enzymatic activities of the nanozyme ZIF-8@PDA@Pt, the catalytic dynamics was investigated. We carried out a steady-state kinetic study to obtain the rate constants by varying the concentration of TMB with a constant concentration of H_2_O_2_ or *vice versa*. The Michaelis constant (*K*_*m*_) and maximum reaction rate (*V*_max_) were calculated from the fitted Michaelis‒Menten curves and Lineweaver‒Burk double reciprocal plots (Figures 2C–F). As a comparison, the obtained *K*_*m*_ and *V*_max_ of ZIF-8@PDA@Pt and other catalysts including the natural enzyme HRP and Fe_3_O_4_ nanozymes are listed in Supplementary Table S1. *K*_*m*_ indicates the enzyme affinity toward substrates, and *V*_max_ defines the maximal catalytic capacity of an enzyme under specified conditions. Generally, a smaller *K*_*m*_ demonstrates higher affinity, while the higher *V*_max_ refers to the better catalytic capability. These results indicated that the obtained nanozyme ZIF-8@PDA@Pt shows better catalytic performance than the classical POD nanozyme Fe_3_O_4_, and some parameters are even significantly better than natural enzymes. For example, *K*_*m*_ of ZIF-8@PDA@Pt was 0.062 mM for TMB, which is much lower than that of HRP and Fe_3_O_4_, indicating that ZIF-8@PDA@Pt has the highest enzyme affinity toward TMB. Moreover, ZIF-8@PDA@Pt also shows the highest *V*_max_ values. These results demonstrated that the obtained ZIF-8@PDA@Pt possesses excellent POD-like activity, also implying the strong ROS generation ability of the ZIF-8@PDA@Pt–H_2_O_2_ system.

**FIGURE 3:**
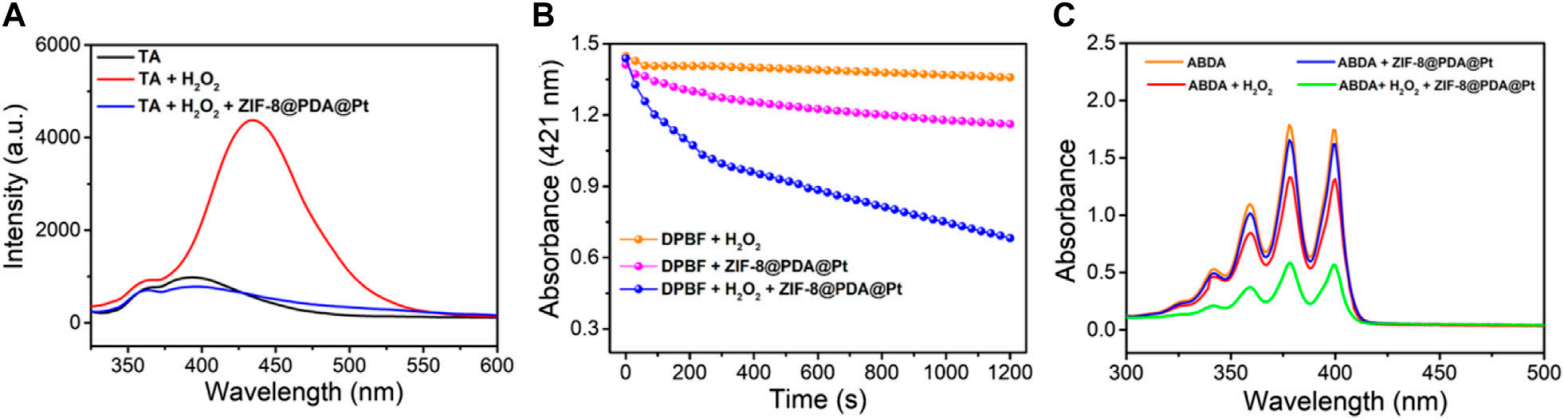
The spectral signals measured by using different ROS probes, TA **(A)**, DPBF **(B)** and ABDA **(C)**.

To further verify the ability of ZIF-8@PDA@Pt to catalyze the production of different free radicals from H_2_O_2_, terephthalic acid (TA), 1,3-diphenylisobenzofuran (DPBF), and 9,10-anthracenediyl-bis(methylene)-dimalonic acid (ABDA) were chose as the indicators for the sensing of hydroxyl radical (•OH), O_2_^•−^ (superoxide anion), and singlet oxygen (^1^O_2_), respectively. TA is a non-fluorescent molecule that can be oxidized by •OH to generated blue fluorescence. DPBF is a fluorescent molecule which possesses a specific reactivity toward ^1^O_2_ and O_2_^•−^, forming an endoperoxide that can decompose to give 1,2-dibenzoylbenzene. This decomposition of DPBF by ^1^O_2_ and O_2_^•−^ can be measured by the decrease in the absorbance intensity of DPBF at 412 nm. ABDA is a highly selective probe for singlet oxygen ^1^O_2_, which can be oxidized with the decrease in the absorbance intensity. As shown in Figure 3, in the solution with the probes and H_2_O_2_, the presence of ZIF-8@PDA@P can induce significant sensing signals of •OH, O_2_^•−^, and ^1^O_2_. These results strongly demonstrated that ZIF-8@PDA@Pt can catalyze the production of different free radical species from H_2_O_2_, which is very important for bacterial disinfection.

### 3.3 Photothermal effect of ZIF-8@PDA@Pt

**FIGURE 4:**
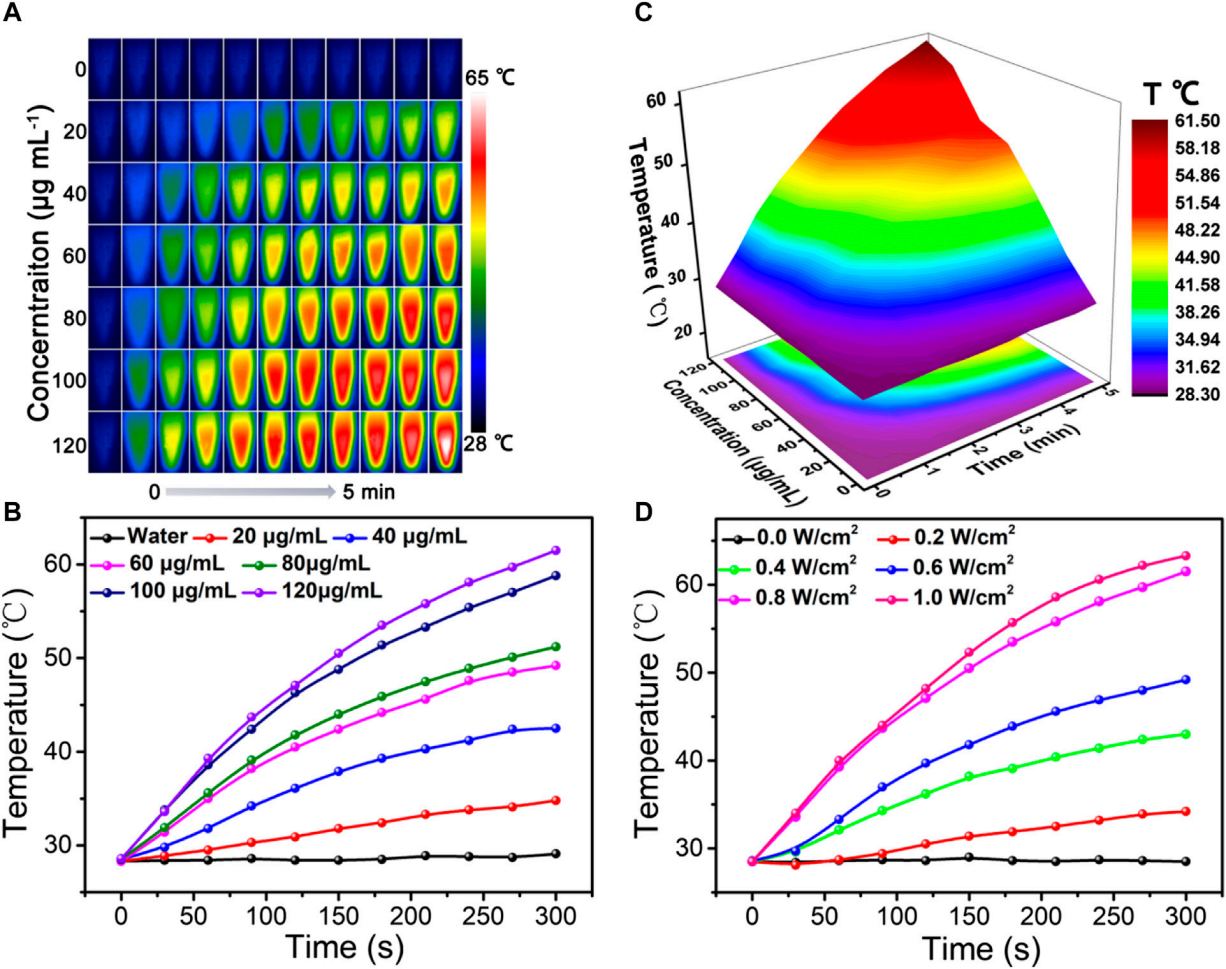
Thermal imaging **(A)**, temperature variation curve **(B)**, and corresponding 3D surface graph **(C)** of ZIF-8@PDA@Pt with different concentrations under laser irradiation (0.8 W) over time. **(D)** Temperature variation curve of the ZIF-8@PDA@Pt solution under different laser intensities over time (808 nm, 120 μg mL^−1^).

As illustrated in the UV-vis spectra of Figure 1F, ZIF-8@PDA@Pt presents broad and intense adsorption from the ultraviolet to NIR region, manifesting ZIF-8@PDA@Pt possesses considerable photothermal conversion potential. So as to validate the supposition, the photothermal property of ZIF-8@PDA@Pt was primarily investigated, where the aqueous dispersions of ZIF-8@PDA@Pt with different concentrations were irradiated under an 808-nm NIR laser. Simultaneously, the real-time temperatures were recorded by a thermal imaging camera. It can be observed from Figures 4A–C that the temperature of the aqueous solution lifted promptly with the augment of the concentration of ZIF-8@PDA@Pt and the prolongation of irradiation time. Indeed, the constant arising trend of the curve implied that ZIF-8@PDA@Pt exhibits supreme photostability under the irradiation of the NIR laser. It can also be visually discovered from the quantitative data that the temperature of the ZIF-8@PDA@Pt solution (120 μg mL^−1^) elevated rapidly from 28.5°C to 61.5°C within 5 min of irradiation, while the temperature of water only raised by 0.8°C under the same conditions. Such a result elucidated ZIF-8@PDA@Pt can serve as a remarkable photothermal agent. Hence, we further calculated the photothermal conversion efficiency of ZIF-8@PDA@Pt in line with a method reported previously (Supplementary Figure S2), where it can be up to 65.4% to our surprise, and this excellent property just met the experimental results we obtained as well, where the real-time temperature of the NIR-irradiated ZIF-8@PDA@Pt aqueous solution can reach up to 61.5°C in a short time.

In addition, it was found that ZIF-8@PDA@Pt presented a laser power-dependent behavior. As shown in Figure 4D, the temperature of solutions at the same time nodes increased gradually along with the increase in laser power, denoting the laser power to be imperative to the photothermal conversion ability of ZIF-8@PDA@Pt. Afterward, the thermal stability of ZIF-8@PDA@Pt was estimated by photothermal performance cycle monitoring within four laser cycles. It can be noted from Supplementary Figure S3 that there were no unmistakable temperature fluctuations in the four photothermal cycles, demonstrating ZIF-8@PDA@Pt endows considerable photothermal stability. All above results certified that ZIF-8@PDA@Pt possessed excellent photothermal properties, which can support its application for sterilization.

### 3.4 Synergistic antibacterial effect of ZIF-8@PDA@Pt

**FIGURE 5:**
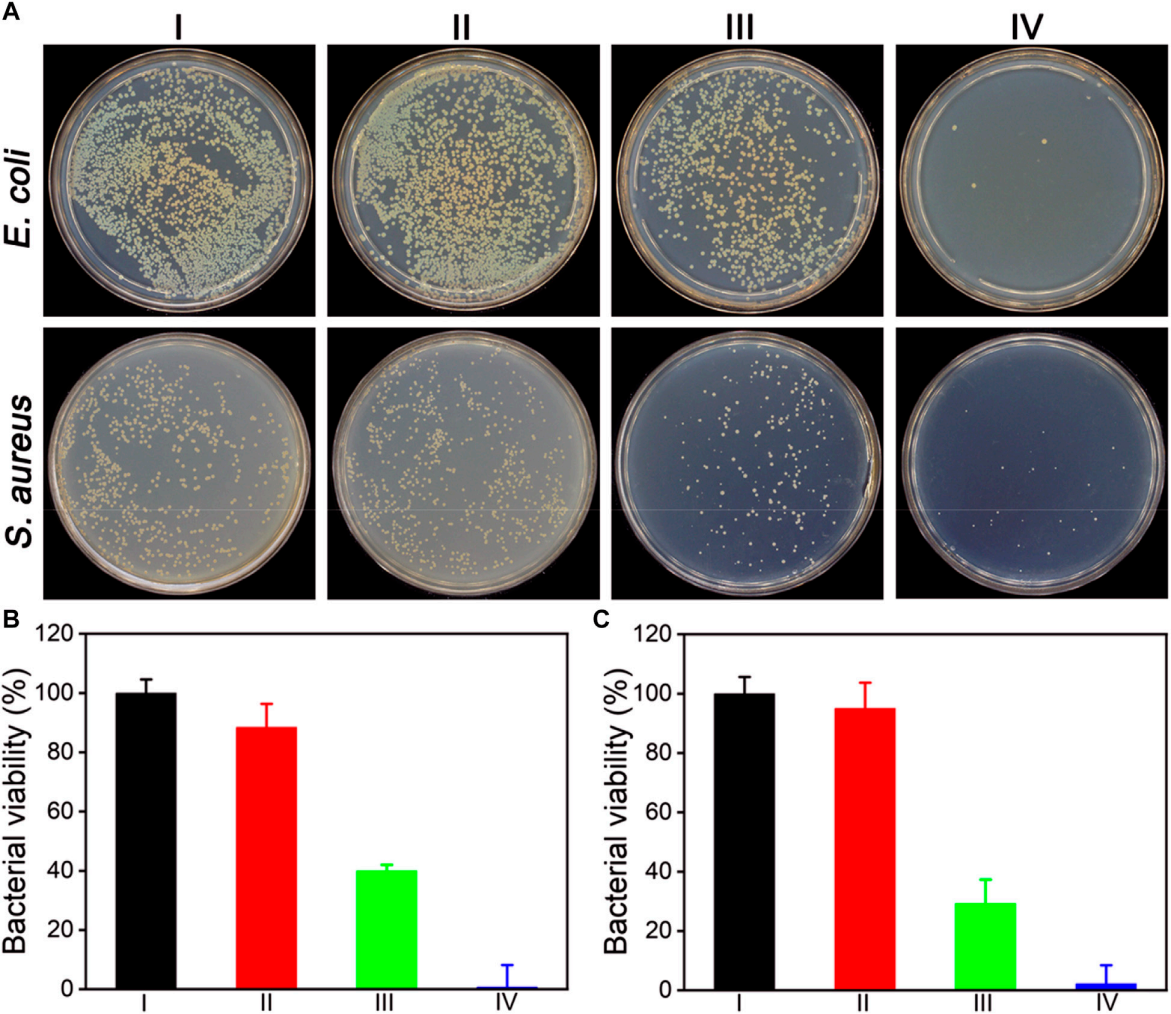
**(A)** Photographs of the colonies of *E. coli* and *S. aureus* treated under different conditions. alculated bacterial viabilities of C*E. coli* **(B)** and *S. aureus* **(C)** in **(A)**. I: Control, II: H_2_O_2_ (2 mM), III: ZIF-8@PDA@Pt + H_2_O_2_, and IV: ZIF-8@PDA@Pt + H_2_O_2_ + NIR. (H_2_O_2_ 2 mM, ZIF-8@PDA@Pt 100 μg mL^−1^, NIR 808 nm 0.8 W 5 min).

**FIGURE 6:**
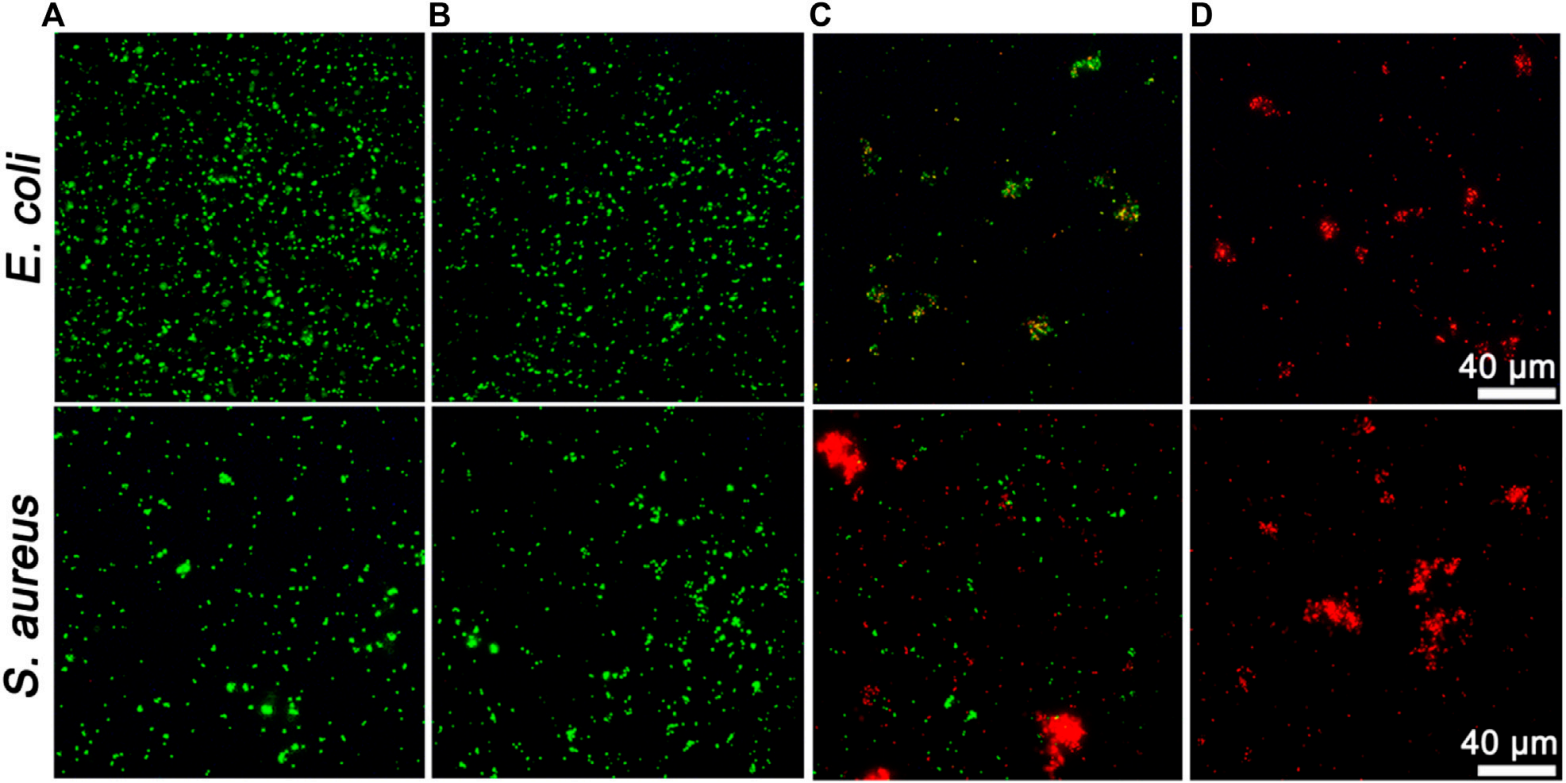
Fluorescent images of live/dead staining of bacteria upon different treatments. **(A)**: Control, **(B)**: H_2_O_2_ (2 mM), **(C)**: ZIF-8@PDA@Pt + H_2_O_2_, and **(D)**: ZIF-8@PDA@Pt + H_2_O_2_ + NIR. (H_2_O_2_ 2 mM, ZIF-8@PDA@Pt 100 μg mL^−1^, NIR 808 nm 0.8 W 5 min).

Inspired by the superior enzymatic activity and photothermal property of ZIF-8@PDA@Pt, the *in vitro* synergistic antibacterial effect of ZIF-8@PDA@Pt against Gram-negative bacteria (*E. coli*) and Gram-positive bacteria (*S. aureus*) was assessed by the plate counting method. As shown in Figure 5, H_2_O_2_ (2 mM) alone cannot induce significant influence on the bacterial viability. However, in the presence of ZIF-8@PDA@Pt, the viabilities of both *E. coli* and *S. aureus* decreased to below 40%. The ROS generated form H_2_O_2_ under the catalysis of ZIF-8@PDA@Pt would be responsible for this sharp decrease in the bacterial viability. Moreover, as expected, NIR irradiation greatly promoted the antibacterial efficiency of the ZIF-8@PDA@Pt/H_2_O_2_ system, in which the bacterial viabilities decreased to below 3%. The viability of the treated bacteria was also investigated by a live/dead staining kit, which contains two dyes, SYTO 9 and propidium iodide (PI). SYTO 9 is a membrane-permeable dye that can give a green fluorescence to indicate the live cells due to the binding with bacterial nucleic acid. PI is also a nucleic acid-binding dye used to give a red fluorescence, which can only penetrate the damaged membrane of dead bacteria. As shown in Figure 6, upon treating with ZIF-8@PDA@Pt and H_2_O_2_, significant amount of dead bacteria was found in the fluorescence images, and almost all of the bacteria were killed when the ZIF-8@PDA@Pt/H_2_O_2_ system was irradiated with the NIR light. This phenomenon was the same as that found in the plate counting experiments. These results clearly demonstrated the high synergistic antimicrobial activity of the nanozyme ZIF-8@PDA@Pt in the presence of H_2_O_2_ and NIR light irradiation. Notably, the nanozyme also has good biocompatibility; in the MTT assay, over 90% of the HSF cells remain alive when in the incubation with 250 μg mL^−1^ of ZIF-8@PDA@Pt.

## 4 Conclusion

In conclusion, a platinum nanozyme ZIF-8@PDA@Pt with high peroxidase-like activity was successfully synthesized. The catalytic activity is much higher than the classical nanozyme Fe_3_O_4_, and some parameters are even better than the natural enzyme HRP. The obtained nanozyme ZIF-8@PDA@Pt can efficiently promote the generation of different ROS from H_2_O_2_. Moreover, ZIF-8@PDA@Pt can harvest the photon energy from the NIR light to heating the solution. The combined excellent photothermal effect and peroxidase-like activity resulted in high synergistic antimicrobial activity of the nanozyme ZIF-8@PDA@Pt in the presence of H_2_O_2_ and NIR light irradiation. The developed nano-antibacterial strategy is highly promising for wide applications, for example, wound infection treatment, antibacterial coatings for medical materials, preservation and protection of agricultural products, etc.

## Data Availability

The original contributions presented in the study are included in the article/Supplementary Material; further inquiries can be directed to the corresponding authors.
