# Supplementary material for: Near-infrared light-heatable platinum nanozyme for synergistic bacterial inhibition
Source: Front Bioeng Biotechnol. 2024 Jan 16;12:1355004. doi: 10.3389/fbioe.2024.1355004 (PMC10824886; doi:10.3389/fbioe.2024.1355004)
Supplement: Supplementary file 1 [file DataSheet1.docx]

Supplementary Material


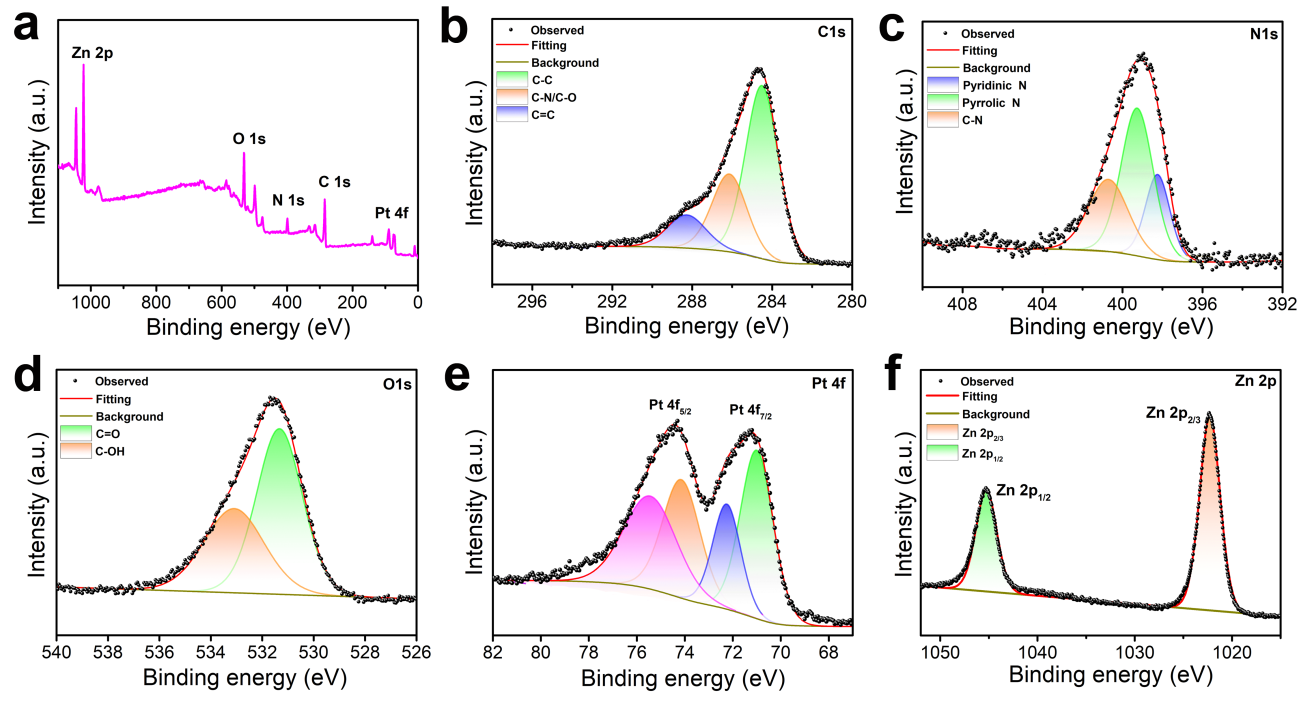


Figure S1 XPS spectrums of ZIF-8@PDA@Pt.


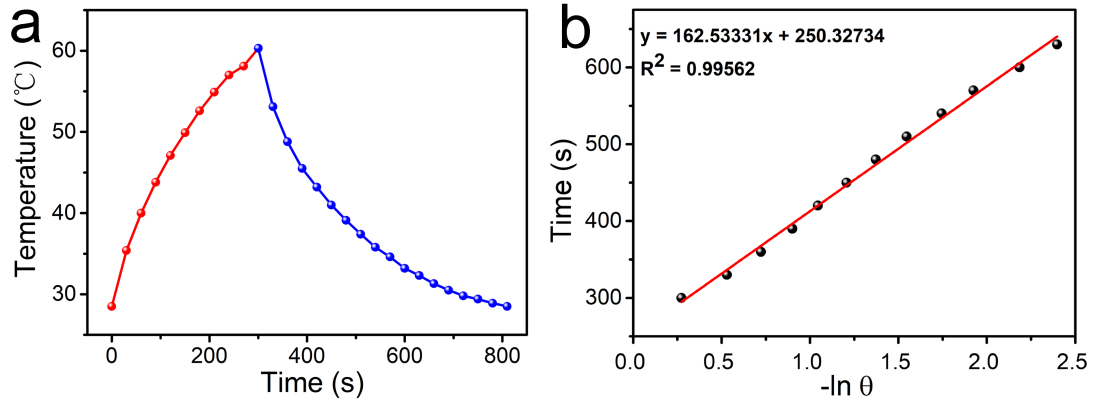


Figure S2 (a) ‘‘On–off’’ temperature change of ZIF-8@PDA@Pt solution (150 μg mL^-1^) under 808 nm laser irradiation (0.8 W). (d) Linear cooling time data versus Ln(*θ*) vs. negative natural logarithm of driving force temperature with *τ*_s_ = 162.53331 s.


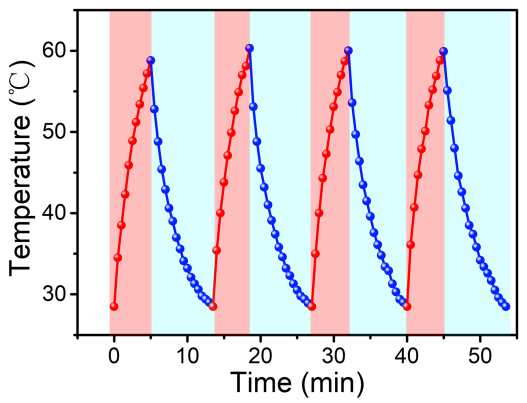


Figure S3 Temperature changes of ZIF-8@PDA@Pt solution during four laser irradiation cycles.


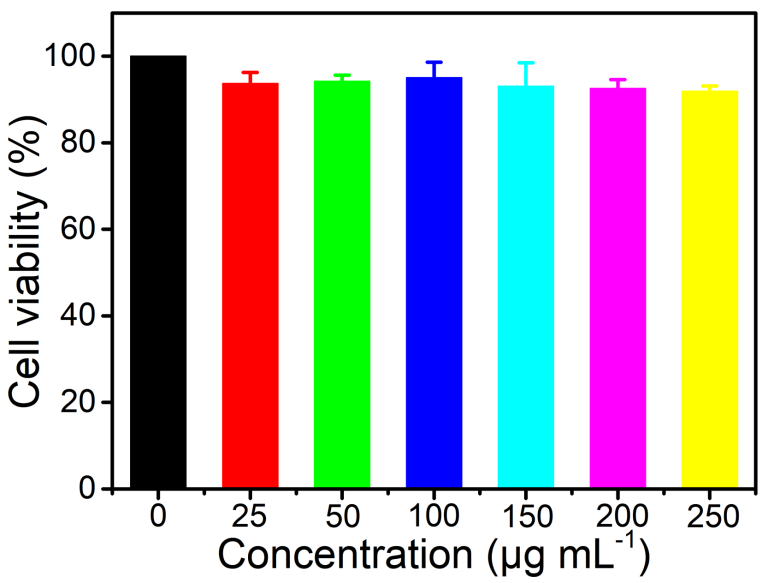


**Figure S4** Cell viability of HSF cells treated with different concentrations of ZIF-8@PDA@Pt solution.

Table S1 Comparison of the apparent Michaelis–Menten constant (*K_m_*) and maximum reaction rate (*V_max_*) of different nanozymes and HRP.

| Catalysts | Substrate | *K_m_* (mM) | *V_max_* (10^-8^ M s^-1^) |
| --- | --- | --- | --- |
| ZIF-8@PDA@Pt | H_2_O_2_ | 6.91 | 286 |
| ZIF-8@PDA@Pt | TMB | 0.062 | 41.7 |
| HRP | H_2_O_2_ | 3.7 | 8.71 |
| HRP | TMB | 0.434 | 10 |
| Fe_3_O_4_ | H_2_O_2_ | 154 | 9.78 |
| Fe_3_O_4_ | TMB | 0.098 | 3.44 |
